# P-1602. SARS-CoV-2 Reinfection and Antibody Kinetics Among Healthcare Workers and Community Members in Ethiopian: A Two-Year Longitudinal Study

**DOI:** 10.1093/ofid/ofaf695.1781

**Published:** 2026-01-11

**Authors:** Esayas Kebede Gudina, Eyob Girma, Rebecca Kisch, Kira Elsbernd, Solomon Ali, Wondimagegn Adissu, Christof Geldmacher, Céline Pellaton, Andreas Wieser, Arne Kroidl

**Affiliations:** Jimma University Institute of Health, Jimma, Oromiya, Ethiopia; Jimma University Institute of Health, Jimma, Oromiya, Ethiopia; Institute of Infectious Diseases and Tropical Medicine, LMU University Hospital, LMU Munich, Munich, Bayern, Germany; Institute of Infectious Diseases and Tropical Medicine, LMU University Hospital, LMU Munich, Munich, Bayern, Germany; St Paul's Hospital Millennium Medical College, Addis Ababa, Adis Abeba, Ethiopia; Jimma University Institute of Health, Jimma, Oromiya, Ethiopia; Institute of Infectious Diseases and Tropical Medicine, LMU University Hospital, LMU Munich, Munich, Bayern, Germany; Division of Immunology and Allergy, Lausanne University Hospital (CHUV) and University of Lausanne, Lausanne, Switzerland, Lausanne, Vaud, Switzerland; Head of Parasitology, Max von Pettenkofer-Institut for Hygiene and Medical Microbiology, LMU Munich, Munich, Bayern, Germany; Division of Infectious Disease and Tropical Medicine, University Hospital (LMU), Munich, MUNICH, Bayern, Germany

## Abstract

**Background:**

SARS-CoV-2 remains a significant public health issue in Ethiopia, where data on reinfection rates, immune responses, and transmission patterns remain limited. This study aimed to analyse antibody responses, reinfection rates and their determinants, and evaluate the seasonality of SARS-CoV-2 infections in Ethiopia.

**Table 1:**
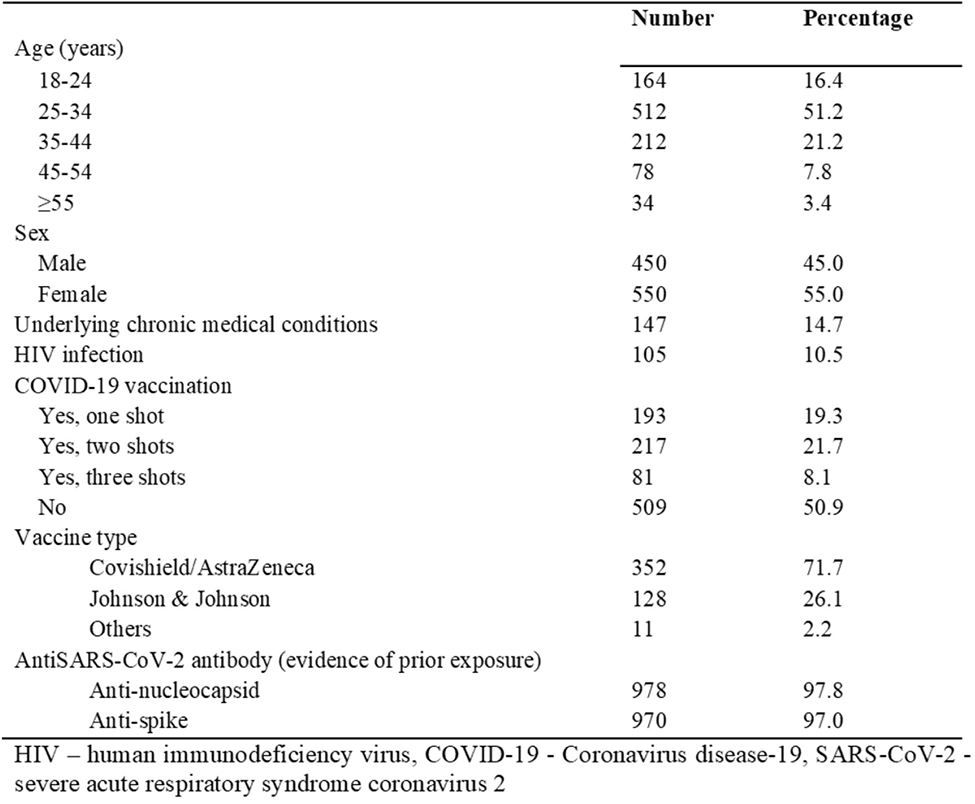
Baseline characteristics of study participants

**Figure 1:**
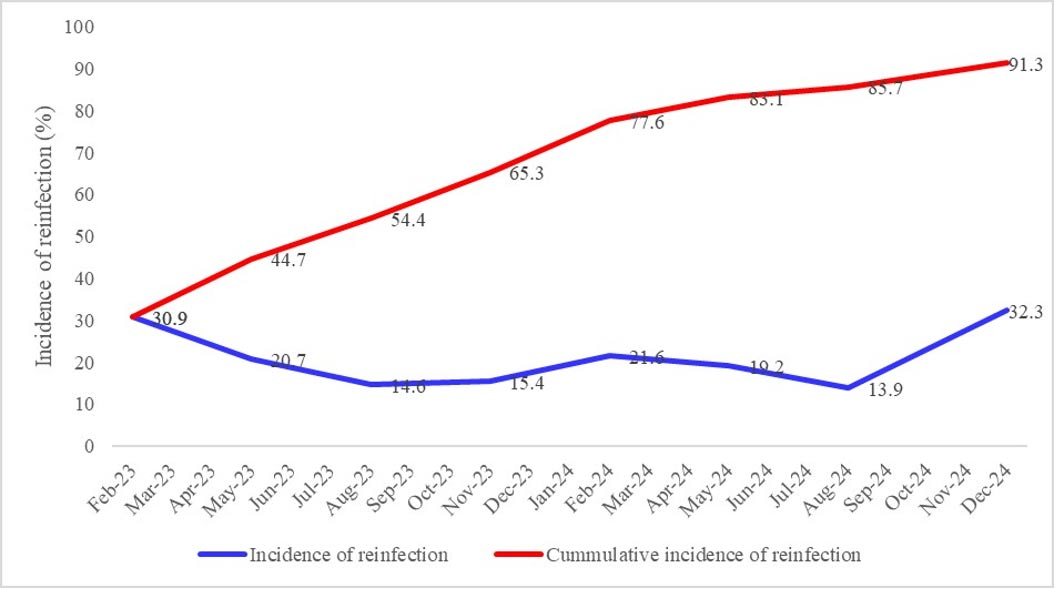
SARSCoV-2 reinfection rate among healthcare workers and community members in Ethiopia (October 2022 to December 2024).

**Methods:**

A longitudinal study was conducted from October 2022 to December 2024 involving 500 healthcare workers (HCWs) and 500 community members. Data collection included baseline questionnaires, quarterly SARS-CoV-2 anti-nucleoside and anti-spike antibody monitoring, annual Interferon-Gamma Release Assays, and neutralization assays against various variants. Reinfection was defined serologically. Nasopharyngeal swab of those with symptoms was tested for respiratory pathogens using Multiplex Real-Time PCR.

**Results:**

Only 49.1% of the participants have ever received COVID-19 vaccine. At baseline, 97.8% of the participants had evidence of prior SARS-CoV-2 infection. Reinfection was frequent, with peaks observed during months of December to February each year. Overall, 91.3% of the participants were reinfected at least once during the follow-up. HCWs exhibited lower odds of reinfection compared to community members (OR=0.73; 95% CI: 0.585–0.907; p=0.005). Vaccination lowered reinfection risk by ∼30%, with 2-dose (OR=0.7; 95% CI: 0.537–0.900; p=0.006) and 3-dose (OR=0.68; 95% CI: 0.478–0.971; p=0.034) recipients having reduced odds compared to unvaccinated individuals. Neutralization assays indicated that antibody responses varied by SARS-CoV-2 variant, vaccine type, number of doses, and history of reinfection. Individuals experiencing reinfection showed higher subsequent neutralization activity, particularly against newer variants. Neutralizing activity generally increased over the 2-year study period, likely due to repeated infections.

**Conclusion:**

This study reveals high SARS-CoV-2 reinfection rates with some seasonal variability. Vaccination provides protection against reinfection. The findings indicate the need for enhanced surveillance, continued vaccination, particularly for high-risk groups, and robust pandemic preparedness strategies tailored to the Ethiopian context.

**Disclosures:**

All Authors: No reported disclosures

